# Construction of prediction models for prolonged length of postoperative hospital stay in patients undergoing primary total hip arthroplasty via direct anterior approach

**DOI:** 10.3389/fsurg.2025.1720930

**Published:** 2026-01-20

**Authors:** Linjie Hu, Guosong Xu, Weiyi Chen, Yiqun Chen, Qichao Ou, Zhibin Wu, Guoxian Chen

**Affiliations:** 1 Department of Orthopaedics, The First Hospital of Putian City, Putian, Fujian, China; 2 The Graduate School of Fujian Medical University, Fuzhou, Fujian, China

**Keywords:** direct anterior approach, length of stay, nomogram, risk factors, total hip arthroplasty

## Abstract

**Purpose:**

To investigate the risk factors associated with prolonged postoperative length of stay (PLOS) in patients undergoing primary total hip arthroplasty (THA) via direct anterior approach (DAA) and develop a perioperative dynamic prediction nomogram for optimizing the perioperative management of THA.

**Methods:**

This single-center, retrospective cohort study analyzed the perioperative clinical data of patients who underwent primary THA through DAA by a single surgical team at our institution between September 2022 to September 2024. Patients were divided into two groups based on postoperative hospital stay duration: the normal group (PLOS < 6 days) and the prolonged group (PLOS > 6 days). LASSO regression was used to screen variables, multivariate logistic regression was applied to establish the model and then a nomogram was developed. The area under the curve (AUC) of receiver operating characteristic (ROC) curve, calibration curve, and decision curve analysis (DCA) were adopted to evaluate the performance and clinical applicability of the model.

**Results:**

This study included a total of 413 patients. Multivariate logistic regression analysis revealed that higher body mass index (BMI), longer operation time, American Society of Anesthesiologists classification (ASA) > II, postoperative extra opioid use, postoperative nausea and vomiting (PONV), postoperative blood transfusion, lower preoperative albumin (ALB) levels, and no prior contralateral THA history were independent risk factors for prolonged postoperative hospital stay in patients undergoing primary DAA-THA (*P* < 0.05). The AUC of the established predictive model was 0.766, indicating good predictive performance. The calibration curve demonstrated good consistency between actual delayed discharge rates and predicted probabilities. DCA showed that the model provided maximum net benefit when the threshold probability ranged from 2% to 85%.

**Conclusions:**

BMI, operation time, ASA classification, postoperative extra opioid use, PONV, postoperative transfusion, preoperative ALB, and previous contralateral THA history can be used as predictive factors. The LASSO regression-based model for predicting prolonged hospital stay after primary DAA-THA demonstrates accurate predictive performance and strong clinical utility. It can assist clinicians in stratifying patient risk effectively, thereby supporting enhanced recovery protocols.

## 1 Introduction

Total hip arthroplasty (THA) is considered one of the most successful surgical innovations of the twentieth century (1). Owing to its excellent clinical outcomes and high patient satisfaction, it remains the gold standard treatment for end-stage hip disease. The direct anterior approach (DAA) utilizes the natural intermuscular plane between the tensor fasciae latae and sartorius muscles (the Hueter interval) to access the hip joint (2). This technique minimizes soft tissue trauma, facilitates early postoperative mobilization, enhances recovery, and can reduce the length of hospital stay (3). Over the past decade, the adoption of minimally invasive surgical (MIS) techniques and the principles of Enhanced Recovery After Surgery (ERAS) in arthroplasty have facilitated a substantial increase in the performance of same-day joint replacement procedures. It is projected that by 2026, over half of all primary THA procedures in the United States will be performed in an outpatient setting (4). Same-day discharge management for selected patients undergoing primary THA has been demonstrated to be a safe, efficient, and cost-effective approach (5–7). Consequently, the utilization of same-day THA programs has shown a steady annual increase (8). However, same-day discharge hip arthroplasty has not yet been widely adopted in China. Currently limited to a few large medical institutions, its implementation lacks standardized protocols, which hinders quality control and widespread dissemination of this practice.

Our institution performed its first same-day discharge DAA-THA in January 2020. While minimizing surgical invasion, reducing pain, and accelerating recovery are central to our department's clinical objectives, a subset of patients continues to experience a significantly prolonged postoperative length of stay (PLOS). A prolonged postoperative hospital stay is frequently associated with the occurrence of perioperative adverse events. This not only increases the utilization of healthcare resources but may also adversely affect the patient's rehabilitation process (9). Currently, a validated tool for assessing the risk of extended hospitalization following primary DAA-THA is lacking. We developed a pragmatic predictive model by analyzing clinical data from our institution. This study aims to identify the key risk factors for prolonged stay after DAA-THA, with the goal of optimizing perioperative management and informing the development of same-day THA programs in China.

## 2 Materials and methods

### 2.1 Study population

This single-center retrospective study analyzed data from the Orthopedic Department records of Putian First Hospital. The cohort included patients who underwent primary DAA-THA between September 2022 and September 2024. Exclusion criteria were: (1) multiple or pathological hip fractures; (2) simultaneous bilateral procedures; (3) use of cemented prostheses; (4) severe hip deformity; (5) poor patient compliance; and (6) incomplete clinical data.

### 2.2 Surgical methods

**Figure 1:**
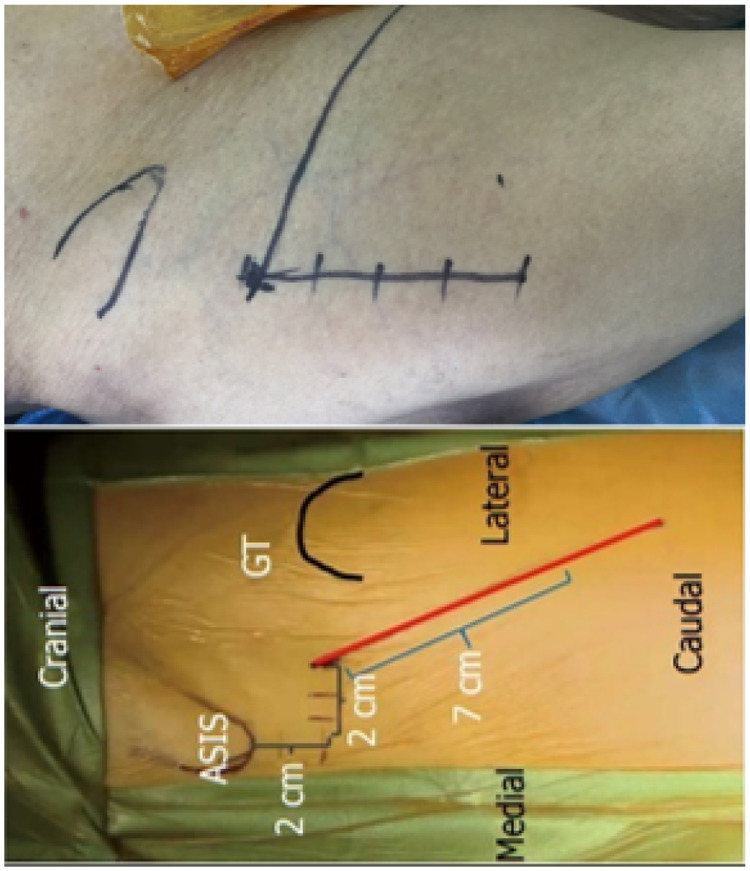
Surgical incision for total hip arthroplasty through the direct anterior approach.

All procedures were performed with the patient in the supine position on a standard folding orthopedic table, with the symphysis pubis aligned to the table's pivot point. A 6 cm–8 cm incision was made along the midline of the tensor fasciae latae muscle, beginning approximately 2 cm distal and 2 cm lateral to the anterior superior iliac spine (Figure 1). All primary THA operations were performed by the same surgical team. The lead surgeon was a senior attending physician with experience in over 300 DAA procedures. The same set of minimally invasive instruments was used for all cases, and a cementless prosthesis (DePuy Synthes, USA) was implanted in every patient.

### 2.3 Perioperative management

A standardized multimodal analgesia protocol was employed. Preoperative administration of non-steroidal anti-inflammatory drugs (NSAIDs) was used for preemptive analgesia. Postoperative pain management consisted of patient-controlled analgesia pumps, scheduled NSAIDs (e.g., celecoxib), and ice therapy. Supplemental opioid analgesics were administered as needed based on individual patient pain assessment. Our perioperative transfusion protocol was initiated on postoperative day 1. Routine blood and biochemical tests were monitored serially. Transfusion was indicated for patients with a hemoglobin level below 80 g/L accompanied by symptoms of anemia, such as palpitations or chest tightness. For asymptomatic patients with similar hemoglobin levels, close observation and serial blood tests were maintained. Patients with hemoglobin levels between 80 and 100 g/L were considered for transfusion if they exhibited unstable vital signs or significant clinical symptoms, including tachycardia (heart rate > 100 bpm), hypotension (systolic blood pressure < 90 mmHg), cardiac chest pain, congestive heart failure, unexplained tachycardia, or hypotension refractory to fluid resuscitation. Patients were deemed ready for discharge upon meeting the following criteria: the ability to ambulate independently and safely on level ground with a walking aid; demonstrated understanding and correct performance of prescribed home exercises (e.g., ankle pumps, quadriceps isometric contractions, straight leg raises) by both the patient and their caregiver; stable vital signs; laboratory values within acceptable limits; a clean, dry surgical incision without signs of infection; adequate pain control; and the absence of other complications.

### 2.4 Data collection

Patient demographic and perioperative data (encompassing preoperative baseline characteristics, intraoperative events, and early postoperative outcomes) were collected from the electronic medical record system. Demographic variables included sex, age, body mass index (BMI), surgical indication (osteoarthritis [OA], femoral neck fracture [FNF], developmental dysplasia of the hip [DDH], osteonecrosis of the femoral head [ONFN]), comorbidities (including hypertension and diabetes), osteoporosis, and history of contralateral THA. Perioperative variables encompassed operation time, anesthesia type (general or combined spinal-epidural), American Society of Anesthesiologists (ASA) physical status classification, postoperative extra opioid use, occurrence of postoperative nausea and vomiting (PONV), postoperative blood transfusion, preoperative and postoperative hemoglobin (Hb) and hematocrit (Hct) levels, preoperative and postoperative albumin (Alb) levels, occurrence of deep vein thrombosis (DVT), femoral stem type (Corail or Tri-Lock BPS), and length of postoperative hospital stay.

### 2.5 Statistical analysis

Statistical analyses were performed using IBM SPSS (version 25.0) and R software (version 4.4.1). Continuous variables are presented as mean ± standard deviation (*X* ± SD), while categorical variables are expressed as proportions or percentages. Group comparisons for continuous variables were conducted using independent samples *t*-tests or Mann–Whitney *U* tests, as appropriate. For categorical variables, Chi-square or Fisher's exact tests were employed. Variables were selected from multiple potential predictors using the Least Absolute Shrinkage and Selection Operator (LASSO) regression method. The model was fitted using the “glmnet” package in R. Prior to analysis, all continuous variables were standardized using *Z*-scores to ensure comparability of regression coefficients. We assessed multicollinearity by calculating the variance inflation factor (VIF < 5) for all candidate predictors. To maintain model simplicity and interpretability, our final model focuses on main effects and does not include interaction terms. Factors identified by LASSO regression were subsequently included in univariate and multivariate logistic regression analyses to control for potential confounding effects. A backward stepwise selection process, guided by minimizing the Akaike Information Criterion (AIC), was employed to identify independent risk factors significantly associated with prolonged postoperative hospital stay. These factors were incorporated into a nomogram based on the multivariate regression analysis. The performance of the predictive model was evaluated using several metrics, including the concordance index (C-index), area under the receiver operating characteristic curve (AUC), calibration curves, and decision curve analysis (DCA). Internal validation was performed using bootstrapping with 1,000 replicates to calculate a corrected concordance index (C-index), ensuring model accuracy and reliability (10). Calibration was assessed by comparing predicted probabilities against observed outcomes, where closer alignment indicated better performance. Finally, DCA was employed to quantify the net benefit across various threshold probabilities, evaluating the clinical utility of the nomogram. A *P*-value of less than 0.05 was considered statistically significant.

## 3 Result

### 3.1 Patient cohort

**Figure 2:**
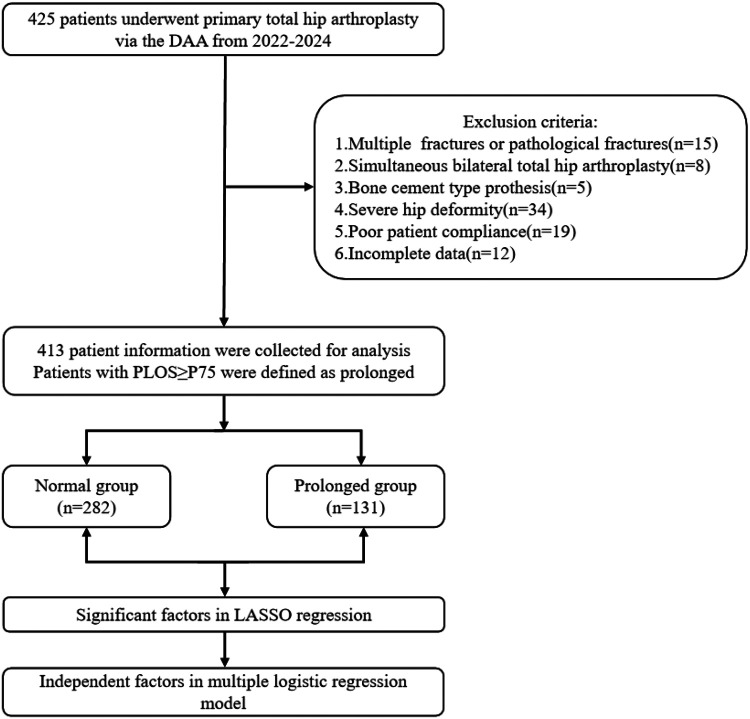
The flowchart.

Based on the predefined inclusion and exclusion criteria, 413 patients were included in the final analysis. The PLOS, the primary outcome measure, was not normally distributed and was therefore described using median and interquartile range (IQR). In the absence of a standard national or international definition for a prolonged hospital stay after DAA-THA, and given the variability in discharge criteria across institutions, we defined prolonged length of stay (PLOS) as a postoperative stay greater than or equal to the 75th percentile of our cohort's distribution. Patients with a PLOS greater than or equal to the 75th percentile of the distribution were defined as the prolonged group (11). Accordingly, 282 patients with a PLOS of less than 6 days (the 75th percentile, P75) were classified into the normal group, while the remaining 131 patients with a PLOS of 6 days or longer comprised the prolonged group (Figure 2). The two groups' perioperative characteristics and complete patient data, including demographics, are shown in Table 1. Between the two groups, statistically significant variations were detected in ASA classification, PONV, postoperative transfusion, prior contralateral THA, preoperative HCT and postoperative ALB (all *P* < 0.001). Compared to patients with normal PLOS, patients with prolonged PLOS were more likely to present higher BMI (*P* = 0.009), longer operative time (*P* = 0.002), postoperative extra opioid use (*P* = 0.005), lower preoperative Hb (*P* = 0.001), and lower preoperative ALB (*P* = 0.006). No significant differences were observed between the two groups in terms of gender, age, type of anesthesia, indications, osteoporosis, DVT, type of prosthesis, postoperative Hb or postoperative HCT (all *P* > 0.05).

**Table 1:** Demographical characteristics and clinical date of prolonged PLOS and normal PLOS groups.

Characteristics	Total (*n* = 413)	Normal (*n* = 282)	Prolonged (*n* = 131)	*P*
Gender				0.764
Male	162 (39.2%)	112 (39.7%)	50 (38.2%)	
Female	251 (60.8%)	170 (60.3%)	81 (61.8%)	
Age (years)	64.72 ± 11.21	64.47 ± 10.49	65.24 ± 12.65	0.515
BMI (kg/m^2^)	23.20 ± 3.42	22.88 ± 3.18	23.89 ± 3.80	0.009
Operation time (min)	74.73 ± 19.92	72.30 ± 16.80	79.95 ± 24.64	0.002
Type of anesthesia				0.644
CSEA	371 (89.8%)	252 (89.4%)	119 (90.8%)	
GA	42 (10.2%)	30 (10.6%)	12 (9.2%)	
ASA classification				<0.001
I–II	402 (97.3%)	280 (99.3%)	122 (93.1%)	
III	11 (2.7%)	2 (0.7%)	9 (6.9%)	
Comorbidity				0.051
No	243 (58.8%)	175 (62.1%)	68 (51.9%)	
Yes	170 (41.2%)	107 (37.9%)	63 (48.1%)	
Indications				0.174
OA	127 (30.8%)	91 (32.3%)	36 (27.5%)	
FNF	68 (16.5%)	52 (18.4%)	16 (12.2%)	
DDH	125 (30.3%)	81 (28.7%)	44 (33.6%)	
ONFH	93 (22.5%)	58 (20.6%)	35 (26.7%)	
Postoperative extra opioid use				0.005
No	384 (93.0%)	269 (95.4%)	115 (87.8%)	
Yes	29 (7.0%)	13 (4.6%)	16 (12.2%)	
PONV				<0.001
No	322 (78.0%)	233 (82.6%)	89 (67.9%)	
Yes	91 (22.0%)	49 (17.4%)	42 (32.1%)	
Postoperative transfusion				<0.001
No	358 (86.7%)	259 (91.8%)	99 (75.6%)	
Yes	55 (13.3%)	23 (8.2%)	32 (24.4%)	
Preoperative Hb (g/L)	130.55 ± 13.75	132.03 ± 13.19	127.38 ± 14.43	0.001
Preoperative HCT (%)	39.42 ± 3.95	39.86 ± 3.84	38.47 ± 4.02	<0.001
Preoperative ALB (g/L)	41.78 ± 3.91	42.14 ± 4.00	41.01 ± 3.60	0.006
Postoperative Hb (g/L)	111.99 ± 14.92	112.96 ± 14.43	109.90 ± 15.78	0.052
Postoperative HCT (%)	33.71 ± 4.40	33.96 ± 4.28	33.16 ± 4.64	0.086
Postoperative ALB (g/L)	35.25 ± 3.12	35.60 ± 3.17	34.50 ± 2.90	<0.001
Osteoporosis				0.975
No	195 (47.2%)	133 (47.2%)	62 (47.3%)	
Yes	218 (52.8%)	149 (52.8%)	69 (52.7%)	
DVT				0.488
No	345 (83.5%)	238 (84.4%)	107 (81.7%)	
Yes	68 (16.5%)	44 (15.6%)	24 (18.3%)	
Type of prosthesis				0.442
Corail	289 (70.0%)	194 (68.8%)	95 (72.5%)	
Tri-Lock BPS	124 (30.0%)	88 (31.2%)	36 (27.5%)	
Prior contralateral THA				<0.001
No	340 (82.3%)	220 (78.0%)	120 (91.6%)	
Yes	73 (17.7%)	62 (22.0%)	11 (8.4%)	

PLOS, postoperative length of stay; BMI, body mass index; GA, General Anesthesia; CSEA, Combined Spinal Epidural Anesthesia; ASA, American Society of Anesthesiologists; OA, osteoarthritis; FNF, femoral neck fracture; DDH, developmental dysplasia of the hip; ONFN, osteonecrosis of the femoral head; PONV, postoperative nausea and vomiting; Hb, hemoglobin; HCT, hematocrit; ALB, albumin; DVT, postoperative deep vein thrombosis; THA, total hip arthroplasty.

### 3.2 Variables selection

**Figure 3:**
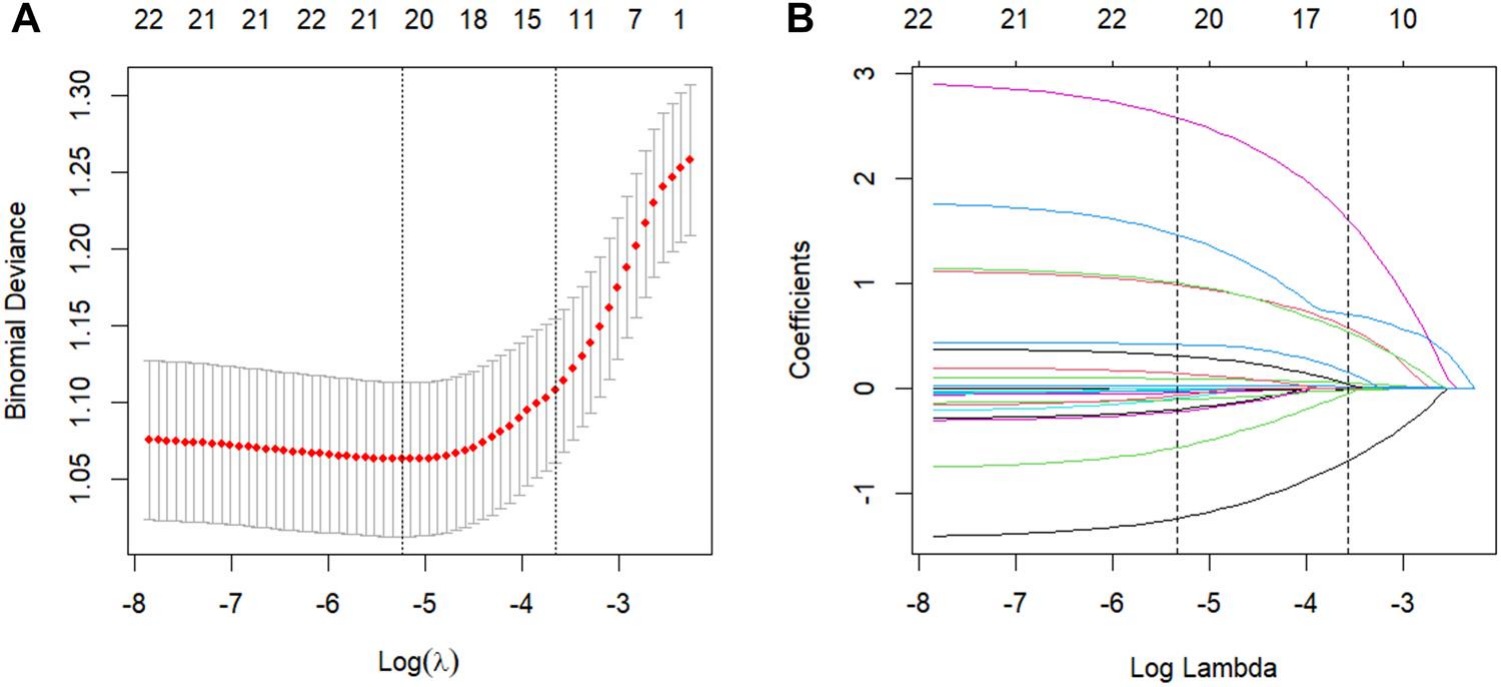
Demographic and clinical feature selection using the LASSO binary logistic regression model. **(A)** Parameter selection in the LASSO model used ten-fold cross-validation via minimum criteria. Partial likelihood deviance (binomial deviance) curves and logarithmic (lambda) curves were plotted. Dotted vertical lines were drawn at the optimal values by using the minimum standard and the 1 SE of the minimum standard (1-SE standard). **(B)** LASSO coefficient profiles of the 24 features. In the present study, predictor's selection was according to the 1-SE standard (right dotted line). LASSO, least absolute shrinkage and selection operator; SE, standard error.

Fourteen variables with non-zero coefficients were selected from 24 initial candidates using LASSO regression with the one-standard-error (1-SE) criterion (Figure 3). These predictors included: BMI, operation time, ASA classification, comorbidity, FNF, ONFH, postoperative extra opioid use, PONV, postoperative transfusion, preoperative Hb and Hct levels, preoperative and postoperative ALB levels, and history of contralateral THA. Univariate analysis was first performed on these 14 predictors, followed by multivariate logistic regression analysis. Ultimately, eight independent risk factors for prolonged PLOS were identified, as presented in Table 2: BMI, operation time, ASA classification, postoperative extra opioid use, PONV, postoperative transfusion, preoperative ALB level, and prior contralateral THA.

**Table 2:** Results of the univariate and multivariate logistic regression analyses for prolonged PLOS.

Variables	Univariate logistic regression analysis		Multivariate logistic regression analysis	
	OR (95% CI)	*P*	OR (95% CI)	*P*
BMI	1.091 (1.026–1.162)	0.006	1.096 (1.024–1.175)	0.009
Operation time	1.019 (1.009–1.031)	<0.001	1.025 (1.012–1.039)	<0.001
ASA classification	10.328 (2.614–68.397)	0.003	18.895 (3.962–145.091)	<0.001
Comorbidity	1.515 (0.997–2.305)	0.052	NA	
Indications				
OA	—	—	—	—
FNF	0.615 (0.328–1.103)	0.115	NA	
DDH	—	—	—	—
ONFH	1.408 (0.864–2.274)	0.165	NA	
Postoperative extra opioid use	2.879 (1.343–6.280)	0.007	3.314 (1.420–7.876)	0.006
PONV	2.244 (1.388–3.625)	<0.001	2.603 (1.504–4.524)	<0.001
Postoperative transfusion	3.640 (2.040–6.590)	<0.001	3.538 (1.821–6.987)	<0.001
Preoperative Hb	0.975 (0.959–0.990)	0.002	NA	
Preoperative HCT	0.911 (0.860–0.962)	0.001	NA	
Preoperative ALB	0.928 (0.878–0.979)	0.007	0.926 (0.870–0.984)	0.014
Postoperative ALB	0.889 (0.827–0.952)	<0.001	NA	
Prior contralateral THA	0.325 (0.157–0.618)	0.001	0.259 (0.116–0.530)	<0.001

BMI, body mass index; ASA, American Society of Anesthesiologists; OA, osteoarthritis; FNF, femoral neck fracture; DDH, developmental dysplasia of the hip; ONFN, osteonecrosis of the femoral head; PONV, postoperative nausea and vomiting; Hb, hemoglobin; HCT, hematocrit; ALB, albumin; THA, total hip arthroplasty.

### 3.3 Nomogram construction and validation for PLOS

**Figure 4:**
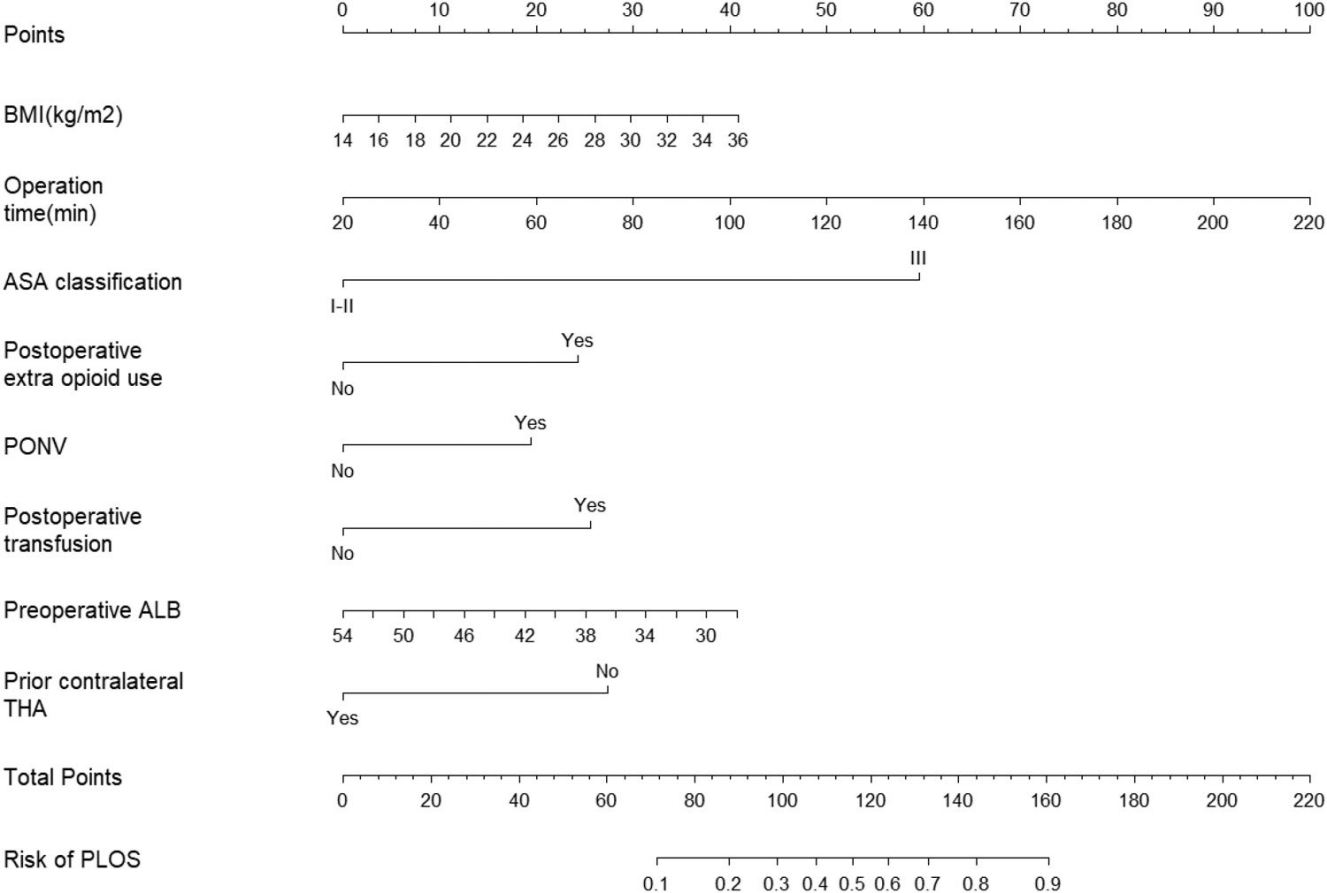
Nomogram for predicting the risk of prolonged postoperative length of stay for primary total hip arthroplasty via DAA.

**Figure 5:**
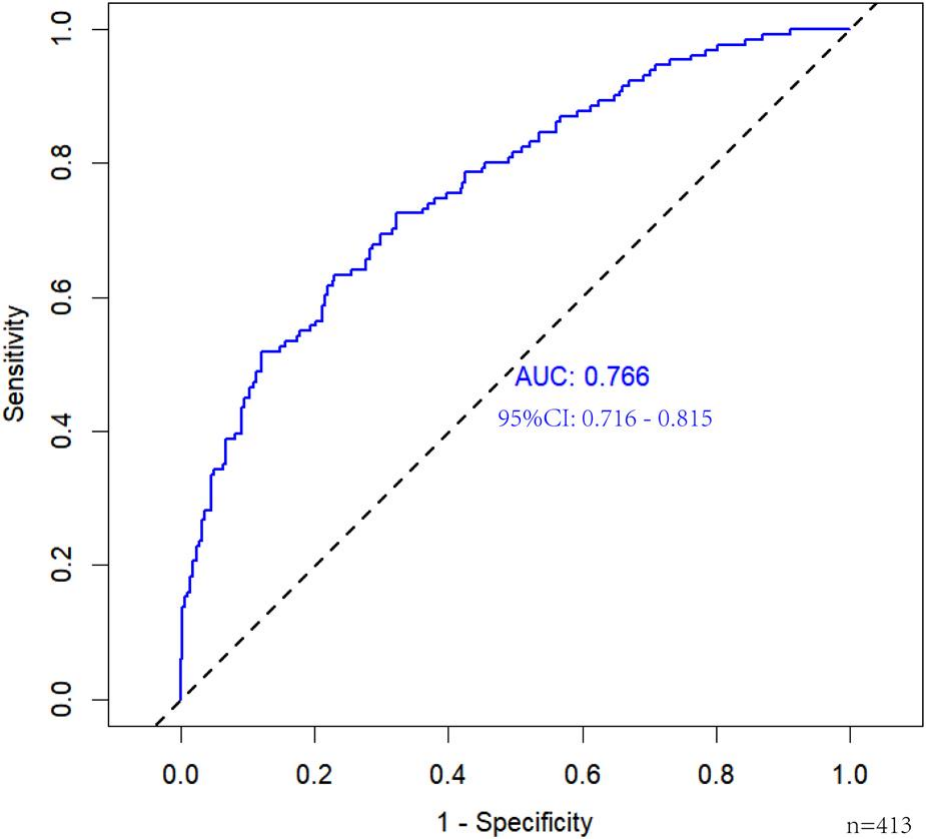
The ROC analysis for the predictive model.

**Figure 6:**
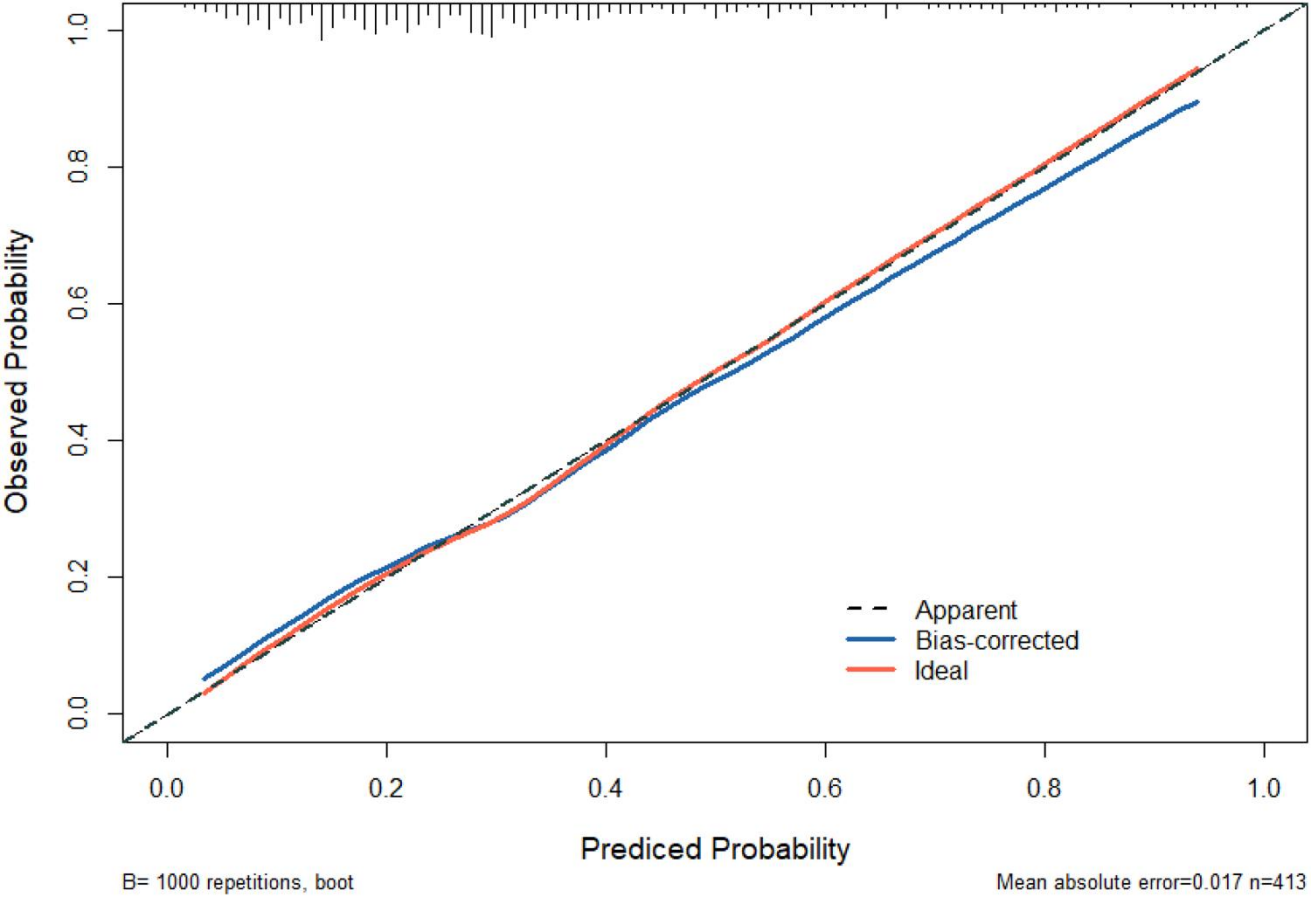
Calibration curve comparing predicted probabilities of prolonged PLOS against observed outcomes. The dashed diagonal line represents the ideal perfect calibration, where predictions exactly match observations. The solid line represents the performance of the nomogram, with closer alignment indicating better predictive accuracy.

The independent risk factors identified through multivariate analysis were incorporated into a predictive model. A nomogram was subsequently developed to visually represent the results of the logistic regression analysis (Figure 4). The nomogram demonstrated good discriminatory ability, with an AUC of 0.766 (95% CI: 0.716–0.815) in the primary cohort. Internal validation using 1,000 bootstrap replicates yielded a corrected AUC of 0.745, confirming the model's robust performance (Figure 5). The calibration curve demonstrated strong agreement between the observed prolonged PLOS and the predicted probabilities (Figure 6).

### 3.4 Clinical application

**Figure 7:**
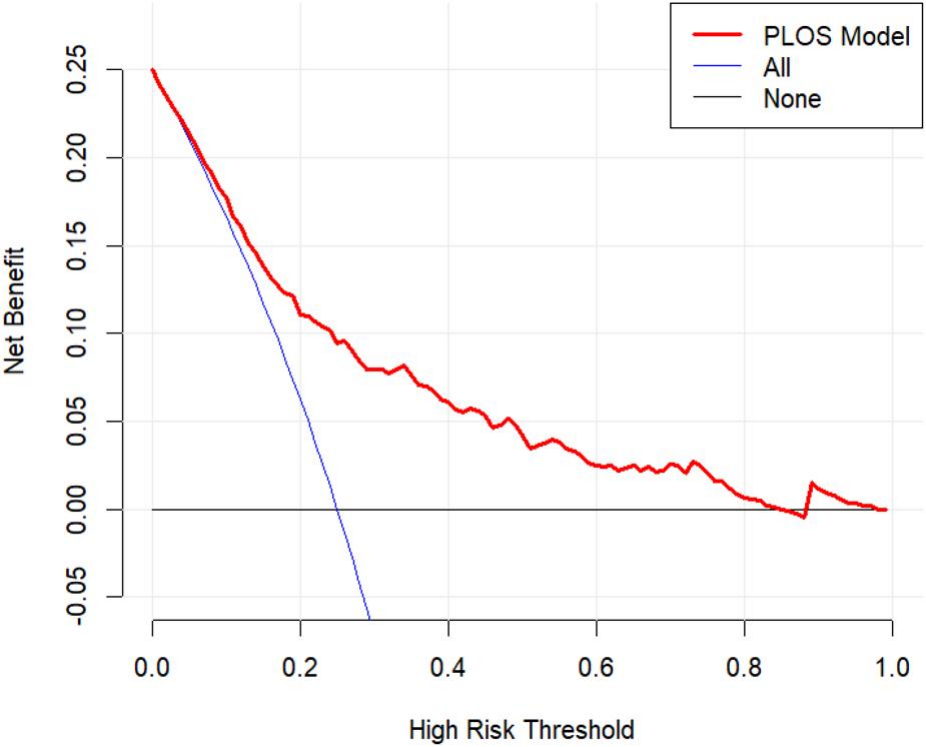
Analysis of the decision curve for the prolonged PLOS prediction model.

DCA was performed to evaluate the clinical utility of the predictive model. This method quantifies the net benefit of using the nomogram across various threshold probabilities. The analysis demonstrated that the model provides a superior net benefit for predicting prolonged PLOS risk at threshold probabilities between 2% and 85% (Figure 7), indicating its clinical value for both clinicians and patients within this range.

## 4 Discussion

Over one million THA procedures are performed globally each year, and this number continues to rise due to an aging population (12). THA has become a highly standardized procedure. With the implementation of Enhanced Recovery After Surgery (ERAS) protocols, the average length of hospital stay for THA has decreased, reducing healthcare costs and accelerating patient recovery (13). Shortening the hospital stay following hip arthroplasty is safe, as current evidence indicates it does not increase the risk of complications or readmission (14–17). For a major procedure like THA, the extent of soft tissue injury significantly influences the pace of recovery. In the early development of same-day THA, minimally invasive surgical approaches were proposed to reduce postoperative complications, with the two-incision technique being one example (18). As minimally invasive techniques have gained popularity, many patients specifically seek surgeons proficient in the DAA (19). The DAA minimizes soft tissue damage and has become the most common technique for same-day THA in recent studies (20). The average length of stay (LOS) is widely used as a composite metric to evaluate healthcare resource utilization, quality of care, and hospital management efficiency (21). The total length of the perioperative hospital stay is influenced by numerous factors. Therefore, the PLOS serves as a more precise indicator of a patient's rate of recovery following surgery. A prolonged PLOS is generally associated with higher costs, increased readmission rates, and more complications (22, 23). Therefore, efforts to reduce the LOS should be pursued, provided that the standard of care is not compromised. This study focuses on identifying risk factors for a prolonged hospital stay following primary DAA-THA. The aim is to optimize perioperative management, facilitate faster recovery, and guide patient selection for same-day THA procedures.

To our knowledge, this is the first study to develop and validate a nomogram for predicting prolonged PLOS following DAA-THA. A meta-analysis by Ramadanov et al. compared DAA-THA outcomes across different surgical table setups. While it found no significant differences in functional scores, radiographic outcomes, or complication rates, it did not evaluate differences in postoperative length of stay (24). The PLOS is influenced by multiple factors, including patient-specific characteristics, surgical variables, and institutional perioperative management protocols. These variations result in significant differences in PLOS across healthcare facilities, thereby limiting direct comparability. In this study, the prolonged postoperative length of stay (PLOS) was defined as a duration greater than or equal to the 75th percentile of the distribution for the included cases, corresponding to ≥6 days. This retrospective study of 413 patients undergoing DAA-THA identified the following independent risk factors for prolonged PLOS: BMI, operation time, ASA classification, postoperative extra opioid use, PONV, postoperative transfusion, preoperative ALB levels, and prior contralateral THA. Based on these eight factors, we developed a visual nomogram that provides an intuitive and accurate prediction of the probability of extended hospitalization following primary DAA-THA. A recent meta-analysis compared the mid- and short-term outcomes of different surgical approaches for total hip arthroplasty, indicating that the SuperPATH approach is associated with superior short-term results. Future work should focus on quantifying differences in predictive models across these various approaches (25).

The association between BMI and outcomes following THA has been extensively studied, yet remains controversial (26–30). Several studies have reported that obesity is associated with higher rates of adverse events, including wound complications, dislocation, revision surgery, and readmission (27, 31). The prolonged hospital stay observed in patients with obesity may be attributable to this increased risk of complications. A retrospective analysis by Hilal et al. of 8,973 patients who underwent THA at the Mayo Clinic between 2000 and 2008 demonstrated that obesity significantly increased both the length of hospitalization and costs, even in the absence of postoperative complications (28). Our study also identified elevated BMI as an independent risk factor for prolonged LOS following primary DAA-THA. Furthermore, it is evident that excess adipose tissue in the surgical field can obscure anatomical landmarks and complicate exposure, thereby increasing procedural difficulty. Consequently, particularly careful perioperative management is warranted for patients with obesity.

THA has become a highly standardized procedure. Operation time and intraoperative blood loss are commonly used as indicators of surgical complexity. Prolonged operative duration has been consistently associated with an increased risk of short-term complications following THA, including surgical site infection, periprosthetic joint infection, and the need for postoperative transfusion (32). Our study similarly found that extended operation time was associated with a higher risk of prolonged PLOS. Therefore, without compromising the quality of the procedure, efforts should be made to minimize operation time to facilitate patient recovery. The ASA Physical Status classification system is widely used to assess a patient's preoperative health and surgical risk. Previous studies have established that patients with more significant comorbidities require a longer postoperative recovery period (26, 33–35). Consistent with this, our study identifies an ASA classification greater than II as an independent factor significantly associated with a prolonged postoperative hospital stay. Therefore, surgeons should prioritize the management of patients with a high ASA classification. Implementing multidisciplinary interventions for these patients can reduce perioperative stress and complications, thereby enhancing recovery.

Effective postoperative pain management is a critical component of perioperative care for THA and a key criterion for patient discharge (36). Multimodal analgesic protocols, which minimize reliance on opioids, have been consistently associated with a reduced length of hospital stay (37–40). This is significant, as opioid use is linked to adverse events that can impede recovery. Evidence suggests that discontinuing opioid use prior to surgery can significantly enhance functional recovery after THA (41). Corroborating this, our study found that additional postoperative opioid requirement was independently associated with a longer hospital stay. Consequently, minimizing perioperative opioid use is recommended to reduce the risk of adverse events and facilitate patient recovery (42). A potential limitation of our model is the reliance on “additional postoperative opioid requirement” as the sole pain-related metric. By not incorporating pain intensity scores or differences in regional analgesic techniques, this measure may not fully capture the impact of postoperative pain on discharge readiness. PONV is a common complication following joint arthroplasty, with reported incidence rates as high as 83% after major orthopedic surgery (43). Beyond patient discomfort, PONV can lead to serious sequelae such as dehydration, electrolyte imbalance, wound dehiscence, and aspiration pneumonia (44). These complications may prolong hospitalization, increase healthcare costs, and ultimately impede recovery. In line with these findings, our study identified PONV as an independent risk factor for delayed discharge following DAA-THA. At our institution, PONV prophylaxis and management are primarily achieved pharmacologically, utilizing agents such as metoclopramide and ondansetron. Patients undergoing THA face a considerable risk of perioperative blood transfusion, with reported rates ranging from 15.9% to 29.8% (45). Perioperative transfusion increases the risk of surgical site infection, thromboembolic events, cardiopulmonary complications, longer hospital stays, higher costs, and even mortality (46, 47). In a retrospective study of 2,104 primary THA patients, Monsef et al. found that allogeneic blood transfusion was associated with prolonged hospitalization, regardless of the number of units transfused (48). Our study further identifies perioperative transfusion as an independent risk factor for extended length of stay following THA, perioperative blood management represents a critical component of enhanced recovery protocols following THA. In our study, preoperative Hb and Hct were significant in univariate analysis but not in the multivariate model. We hypothesize that this may be because postoperative blood transfusion demonstrated a stronger association with prolonged length of stay. Therefore, the primary clinical goal in managing preoperative anemia is to prevent the need for postoperative transfusion.

Furthermore, our analysis identified low preoperative albumin as an independent risk factor for prolonged postoperative hospitalization. Reduced serum albumin decreases colloid osmotic pressure, leading to fluid accumulation in the interstitial space. This may manifest as wound exudate at the surgical site, impairing healing and increasing the risk of perioperative complications. In a retrospective study of 19,975 patients undergoing primary THA, Daniel reported that hypoalbuminemia was associated with a higher incidence of surgical site infection, pneumonia, prolonged LOS, and readmission (49). Therefore, preoperative assessment of serum albumin levels is essential, and nutritional support should be optimized for patients with hypoalbuminemia.

Notably, our study found that patients with a history of contralateral THA had a lower risk of delayed discharge. This may be attributed to differences in patient-specific factors and prior perioperative experience. These patients likely have more realistic expectations, better compliance with perioperative management protocols, and greater familiarity with the rehabilitation process. To leverage this effect, we recommend developing center-specific patient education materials that systematically outline the entire perioperative journey. Such resources would empower patients by optimizing their cognitive, behavioral, and psychological preparedness, thereby facilitating recovery.

## 5 Limitations

This study has several limitations. First, as a single-center retrospective analysis conducted exclusively in a Chinese population with a relatively small sample size, the findings may be subject to selection bias. Second, complete data on perioperative complications were unavailable; some non-mandatory complications, such as transient hypotension, were not systematically recorded. These omissions were primarily random, attributable to variations in documentation practices. Nonetheless, their potential association with PLOS cannot be ruled out. Finally, the use of 6 days as the threshold for prolonged PLOS may limit generalizability, as average hospital stay durations can vary substantially across institutions. Future prospective, multi-center studies with larger sample sizes are needed to validate these results and refine the predictive model.

## 6 Conclusion

This study identified BMI, operation time, ASA classification, postoperative extra opioid use, PONV, postoperative transfusion, preoperative ALB levels, and history of contralateral THA as independent factors associated with delayed discharge following DAA-THA. Based on a comprehensive set of perioperative variables, we developed a nomogram to predict prolonged LOS after primary DAA-THA that demonstrated good predictive accuracy. This tool may assist clinicians in stratifying patient risk, optimizing perioperative management, enhancing surgical safety, facilitating recovery, and informing patient selection for same-day THA procedures.

## Data Availability

The raw data supporting the conclusions of this article will be made available by the authors, without undue reservation. Requests to access these datasets should be directed to Guoxian Chen, cgx15960545510@163.com.
